# Supplementary material for: Comparative multiomics analysis of cell physiological state after culture in a basket bioreactor
Source: Sci Rep. 2022 Nov 23;12:20161. doi: 10.1038/s41598-022-24687-4 (PMC9686226; doi:10.1038/s41598-022-24687-4)
Supplement: Supplementary file 1 — Supplementary Information 1. [file 41598_2022_24687_MOESM1_ESM.zip › raw data/Proteomic raw data/6.KEGGenrichment/EG.vs.CG/DOWN/pathway/src/01521.html]

map01521


- K05464

- Down regulated proteins

A0A0D9RXU0(Description:Growth arrest specific 6;Gene:GAS6;log2FC:-1.0207)

- K05115

- Down regulated proteins

A0A0D9S0W1(Description:Receptor protein-tyrosine kinase;Gene:AXL;log2FC:-1.0495)

Close
